# Editorial: Neuroscience and Neurotechnology of Neuronal Cell Surface Molecules in Neural Circuits

**DOI:** 10.3389/fncir.2021.703300

**Published:** 2021-06-23

**Authors:** Masahito Yamagata, Hiroko Bannai

**Affiliations:** ^1^Faculty of Arts and Sciences, Center for Brain Science, Harvard University, Cambridge, MA, United States; ^2^School of Advanced Science and Engineering, Waseda University, Tokyo, Japan

**Keywords:** extracellular matrix, neurotransmitter receptors, neural circuit, neuronal cell surface molecule, cell adhesion molecule

A myriad of specialized neurons and glial cells interact with each other in the nervous system. In this Research Topic, we focused on the cell surface molecules, which are essential for such interactions in the nervous system. They play roles in the development and regeneration of neural circuits (e.g., membrane receptors, extracellular matrix molecules, adhesion molecules) and the function of neural circuits (e.g., neurotransmitter receptors, ion channels). The structure, number, and localization of these molecules vary in different neurological and psychiatric disorders. Nevertheless, many technical challenges have been slowing down studies on extracellular and transmembrane proteins in modern neuroscience. These molecules are also instrumental in labeling and modifying neural circuits (e.g., genetically encoded synthetic proteins in optogenetics, molecular assemblies for neuronal tracing, reagents for detecting synaptic membrane proteins, and tools for remodeling neural circuits) (Bradbury et al., 2002; Feinberg et al., 2008; Loh et al., 2016; Deisseroth and Hegemann, 2017; Bannai, 2018; Suzuki et al., 2020).

This Research Topic eBook is organized into three chapters covering different aspects of cell surface molecules in the nervous system. The first two chapters illustrate the neuroscience of cell interaction molecules, surface receptors, and other components. In particular, we cover a wide range of topics on the cell interaction molecules in neural circuit formation. The last chapter focuses on neurotechnology using membrane proteins as tools.

In Chapter 1, the reader will find articles related to a series of surface molecules involved in neural circuit formation. Such proteins are structurally diverse but consist of a panoply of common domains. One of the most abundant is the immunoglobulin (Ig) domain. The protein families with this domain constitute Ig superfamilies (IgSF) and participate in various cell interactions in the nervous system. Fibronectin type III (FnIII) domains are cognate to the Ig domain, as both constitute β-sandwiches, and they frequently coexist within the same protein. Pourhoseini et al. and Yamagata describe the roles of such IgSFs in neural circuit formation in the retina. Cadherin (Cdh) and leucine-rich repeat (LRR) also play essential roles in cell adhesion and interaction. Pancho et al. comprehensively review protocadherins (also see Jia and Wu, 2020). Polanco et al. performed computational analysis on the expression of classic cadherins in the nervous system, demonstrating that surface molecules can be studied *in silico*. Tsuboi focuses on TPBG (5T4), an LRR protein, showing its role in the olfactory system. Matsunaga and Aruga describe Elfn proteins, which contain LRR and FnIII domains. Neurexins, classical synaptic organizer proteins, contain epidermal growth factor (EGF) and Laminin-α, Neurexin, and Sex hormone-binding globulin (LNS) and work with some ligands such as neuroligins and the Ig/FnIII receptor tyrosine kinase LAR-RTP. Lee et al. dissected these synaptic organizers in Alzheimer's disease. Other proteins containing collagenous domains and carbohydrates are also vital for synapse formation. Wakabayashi reviews transmembrane collagens during the development of neuromuscular junctions. Briatore et al. report a role of dystroglycan in cerebellar synapse formation. Kamimura and Maeda detail glypicans and heparan sulfate proteoglycans in the nervous system. Burger et al. show that C1q, a complement component, in microglia maintains neural circuits in the retina by the atypical mechanism independent of the C3a receptor (C3aR) and complement receptor 3 (CR3). Of note, some C1q complement family members act as synaptic organizers (Yuzaki, 2017). The reader will also notice that articles in this chapter contain diverse species from humans to worms and various systems such as the visual system (Pourhoseini et al.; Yamagata; Tsuboi; Matsunaga and Aruga; Burger et al.), the olfactory system (Tsuboi), the cerebellum (Briatore et al.), and the neuromuscular junction (Wakabayashi). Decades ago, a handful of cell surface proteins were studied as molecules that serve as basic cell adhesion or axon guidance. The reader will find that this chapter tackles advanced processes such as synapse formation, neurite branching, and gene regulation. Jin and Kim provide an overview of neurite branching in nematodes. Kozlova et al. discuss how neuronal adhesion molecules influence protein synthesis, which may occur during development, and synaptic plasticity.

In Chapter 2, the authors describe the unique functions and expression of diverse cell surface and extracellular molecules in the nervous system. Ni reviews ionotropic glutamate receptors in flies. Jimenez-Trejo et al. used immunochemical techniques to find a serotonin system in the testes. Miederer et al. report their method for observing a cannabinoid type 1 receptor *in vivo*. Iwamoto and Oiki describe the physical features of the membrane using an advanced lipid bilayer platform. Ramani et al. reported the effects of oxygen exposure on the development of hippocampal synaptic circuits.

In Chapter 3, the authors describe cutting-edge circuit-tracing methods using the properties of membrane proteins. Sugiyama et al. introduce a method for studying neural circuits by overexpressing a type II membrane protein VAMP2 reporter by single-cell electroporation. Liao et al. explored a highly sensitive membrane-associated reporter (alkaline phosphatase) to discover new oxytocin-expressing circuits. Pan et al. used a retrograde tracer endocytosed by the membranes at axonal terminals. Finally, Gui et al. evaluated the role of the dopaminergic pathway in general anesthesia by adapting a genetically encoded dopamine sensor, optogenetics, and chemogenetics.

In summary, we hope that this book enables the reader to learn about various aspects of surface molecules in neural circuits.

## Author Contributions

MY and HB wrote the manuscript. All authors contributed to the article and approved the submitted version.

## Conflict of Interest

The authors declare that the research was conducted in the absence of any commercial or financial relationships that could be construed as a potential conflict of interest.
